# Occipital Petalia and Albinism: A Study of Interhemispheric VEP Asymmetries in Albinism with No Nystagmus

**DOI:** 10.3390/jcm8060802

**Published:** 2019-06-05

**Authors:** Alkiviades Liasis, Sian E. Handley, Ken K. Nischal

**Affiliations:** 1 Children’s Hospital of Pittsburgh of UPMC, Pittsburgh, PA 15201, USA; nischalkk@upmc.edu; 2 School of Medicine, University of Pittsburgh, Pittsburgh, PA 15201, USA; 3 UCL Great Ormond Street Institute of Child Health, University College London, London WC1E 6BT, UK; Sian.handley@gosh.nhs.uk

**Keywords:** albinism, visual evoked potential, hemifield, misrouting

## Abstract

The purpose of this study was to assess chiasmal misrouting in a cohort of children with albinism with no nystagmus using hemifield visual evoked potentials (VEP) measures. Methods: Monocular VEPs were recorded and analyzed from three electrodes (O1, Oz, and O2 referred to Fz) from 16 children with albinism without nystagmus. Pattern reversal (full field and hemifield stimulation), full field pattern appearance and flash stimuli were used to evoke VEPs for each eye. Results: The amplitude of the pattern reversal VEPs to stimulation of the hemifield corresponding to the crossing pathways were as expected significantly larger than those to the non-crossing in each eye ((right eye *p* = 0.000004), (left eye *p* = 0.001)). Pattern reversal VEPs recorded from the left hemisphere were also larger than those from the right and most evident when comparing the crossing pathways of each eye (*p* = 0.004). Conclusions: This study has demonstrated electrophysiological differences in visual pathway function of the left and right hemisphere in subjects with albinism like that previously described in controls. Nasal field stimulation activated crossing and non-crossing pathways in patients with albinism and as a result, nasal hemifield VEPs in albinism are less lateralized compared to what is found in normal subjects.

## 1. Introduction

Albinism is a heterogeneous group of genetic disorders characterized by absent or reduced melanin pigment in the eye, skin, and hair [1]. The ophthalmological features of individuals with albinism are fundus hypopigmentation, foveal hypoplasia of varying degrees, iris transillumination, nystagmus, and an abnormal decussation pattern at the optic chiasm [2]. The abnormal pattern of decussation consists of an increased number of ganglion cell axons from the temporal hemiretina, (representing the nasal field), crossing the chiasm together with the normal crossing of fibers from the nasal hemiretina. As a result there is a net increase in the number of retinal fibers from one eye projecting to the contralateral hemisphere [3,4]. The extent of this abnormal decussation in albinism has been shown electrophysiologically to range between 2 to 15 degrees [5]. Similarly, there is a spectrum of the melanin pigmentation in the skin and eye; some individuals have near normal ocular and cutaneous pigmentation, which makes diagnosis challenging. Indeed foveal hypoplasia and optic nerve misrouting can occur independently of defects in the melanin biosynthesis pathway and has been demonstrated in recessive mutations of SLC38A8 [6,7].

Visual evoked potentials (VEPs) are a validated technique for identifying chiasmal dysfunction or disproportional decussation in the clinical environment [8,9,10]. In order to detect chiasmal disproportion, monocular VEPs must be recorded employing a trans-occipital electrode array (clinically three to five electrodes are suggested) [11]. The presence or absence of chiasmal misrouting relies on an interocular comparison of the monocular VEP trans-occipital distribution. If a trans-occipital asymmetry of the VEP from one eye is mirrored across the midline by the other eye it is termed a “crossed asymmetry” [12]. This can be observed if too many fibers cross as in albinism but also in the other extreme in achiasmia where there is no crossing [8,13,14,15].

In VEP studies of patients with albinism, the detection of chiasmal misrouting ranges between 18.75% and 100% depending upon the age of the patient and type of VEP stimulus used [16,17]. In albinism, a trans-occipital crossed asymmetry in flash VEPs (fVEP) is best seen in infancy and declines with increased age, while the pattern appearance VEP (paVEP) is more sensitive above the age of seven and may fail to be diagnosed in younger children [18,19]. Unlike fVEP and paVEPs, the pattern reversal VEP (prVEP) waveform has a characteristic single positive peak from infancy, with a well-defined maturational time course. Peak latencies are within 10% of normal adult values by seven months of age [20] making prVEPs ideal for studies of children across a range of ages, and is predominantly the stimulus used in pediatric and adult practice. 

Pattern appearance and flash stimuli have been typically used in most electrophysiological studies of chiasmal misrouting in albinism as prVEP responses can be confounded by nystagmus, which is present in the majority of people with albinism [16,21]. Although potentially under diagnosed, albinism without nystagmus is relatively rare, occurring in 6% to 11% of people with albinism [22,23]. Monocular hemifield (HF) prVEP stimulation is a sensitive way to identify hemisphere and chiasmal dysfunction in patients with steady central fixation [24,25]. HF prVEPs are also invaluable for determining whether a full field VEP trans-occipital asymmetry is pathological or artifactual due to dipole orientation [10]. 

Anatomically in normal subjects the left and right hemispheres are not symmetrical. In the majority of right handed subjects the left occipital lobe is wider and has a greater volume, protruding and curving over the right hemisphere resulting in the cortical asymmetry termed occipital petalia. There is also frontal petalia where the right frontal lobe curves over the left. The combination of these two hemisphere asymmetries can result in a twisting effect of the cortex, bending the posterior interhemispheric fissure to the right, known as Yakovlevian anticlockwise torque [26]. This is an important factor to consider when analyzing VEP asymmetries. The structurally asymmetric occipital petalia can cause smaller and more symmetrically distributed left HF prVEP and larger right HF prVEPs with a typical transoccipital distribution in normal subjects [10,27,28].

Recently imaging studies have shown how alterations in the retina can result in changes at the cortex. Patients with foveal hypoplasia associated with albinism and aniridia have shorter calcarine fissures [29]. It has also been shown that the underdeveloped central retina leads to a decreased number of ganglion cells, which in turn reduces the width of the optic nerves, chiasm, and tracts [3]. In addition, a reduction in gray matter volume in the posterior occipital cortex associated with less gyrification has been observed in subjects with albinism compared to controls [30]. Another study has revealed further subtle differences in the morphology of the occipital cortex in subjects with albinism compared to controls including: increased cortical thickness across V1, increased gray matter in the calcarine sulcus, and a decrease in gray matter volume in the posterior ventral occipital cortex. The decrease in gray matter volume is thought to be due to a decrease in gyrification over both hemispheres, although to a greater extent over the left hemisphere [4]. 

The purpose of this study was to assess chiasmal misrouting in a cohort of children with albinism and no nystagmus by means of hemifield prVEPs.

## 2. Experimental Section

### 2.1. Materials and Methods

This was a cross sectional study registered with the local research and development office (16HS10) and adhered to the tenets of the Declaration of Helsinki.

### 2.2. Participants

16 patients with a clinical diagnosis of albinism without nystagmus (evident on slit lamp microscope examination in different positions of gaze or under monocular conditions) were included in the study (Table 1). Patients were seen at Great Ormond Street Hospital London. The diagnosis of albinism was based on the clinical features, optical coherence tomography (OCT) of the maculae, and electrophysiology findings. To quantify the extent of foveal hypoplasia detected on OCT, images were graded as per the classification by Mohammed et al. [31].

The cohort consisted of 10 females and 6 males with a mean age of 8.1 years (standard deviation 2.48, range 4.8–13.6). All patients were able to maintain central fixation adequately during electrophysiological testing. A summary of all patients is provided in Table 1.

Visual evoked potential recording procedure: Within the same recording session, up to four VEP studies were conducted in all participants (three using pattern stimuli and one using a flash stimulus). For all pattern VEP experiments the participants were seated comfortably in a darkened room 1 m from the stimulus plasma display screen (Model PDP 433MXE; Pioneer Electronics Corp, Tokyo, Japan), of mean screen luminance of 93 cd/m^2^. All recordings were carried for each eye independently with the eye not being tested occluded with a Coverlet eye patch (BSN Medical). VEPs were recorded from three electrodes (Oz, O1, and O2) using Ag–AgCl electrodes, referred to Fz and the ground electrode at Pz in accordance with the 10–20 electrode location system [11]. The electrodes were applied to the scalp using a conductive cream (Elefix; Nihon Kohden, Tokyo, Japan) after abrasion of the scalp in order to maintain their impedances below 5 KΩ throughout the recordings. The electroencephalogram (EEG) was digitized using a sampling rate of 1 kHz and a band-pass filter of 0.312–100 Hz. The amplifiers had a fixed gain with an input range of ±0.5 V (Espion by Diagnosys, Cambridge, UK). Epochs of 300 ms (−15 to 285 ms) were averaged online with any exceeding ±200 μV automatically rejected to create an individual trial. A minimum of two reproducible trials were recorded for each stimulus. In all cases, the grand average of the acquired trials was analyzed. During the recordings the ongoing EEG was also monitored for slow-wave activity associated with drowsiness while fixation was monitored by a closed-circuit television.

Stimuli: Three pattern stimuli were employed, full field (FF) pattern reversal (prVEP), hemifield (HF) pattern reversal (prVEP), and FF pattern appearance (paVEP). All patterned stimuli consisted of high contrast (Michelson contrast: 97%), black and white check elements presented in a 28 degree test field in accord with the ISCEV recommendations. The individual check elements had a side sub tense of 50 min of arc [11]. The same size test check was used for all types of pattern stimulation. During all prVEP testing the checkerboard reversed three reversals/second. During HF prVEP stimulation, half the screen was masked in a horizontal direction from the vertical midline with a uniform gray background of equal overall mean luminance as the black and white checkerboard. During FF paVEP testing the checkerboard appeared for 230 ms followed by a uniform gray background for 300 ms. A red fixation spot was used in the center of the screen measuring 0.25 degrees for FF, and 0.5 degrees for HF stimulation, which was within the acuity range of children in the study. paVEP stimulation was only carried out in participants who had remained alert. Flash stimuli were evoked by a hand help stimulator (Grass stimulator, Grass Instrument Company, Natus Medical Incorporated, Pleasanton, California, CA, USA) at intensity setting 4 held at 30 cm to the subjects. 

### 2.3. Data Analysis

#### 2.3.1. Full Field Stimuli (Flash, Pattern Reversal, and Pattern Appearance)

The amplitude and latency of the major components were measured at Oz, O1, and O2 and compared across the eyes using paired t-tests. For fVEPs the P2 was measured, for prVEP the P100, and for paVEP the first positive peak (CI). Interhemispheric differences in amplitude for each eye were obtained by subtracting the amplitude of the main positive VEP peak obtained at the right occiput from that on the left occiput and displayed graphically. Group amplitude and latencies of the major components for each stimulus were calculated and tabulated. 

#### 2.3.2. Hemifield Pattern Reversal VEP Stimuli

For each participant eye and HF the P100 component was measured at Oz and over the hemisphere ipsilateral to the eye being stimulated (iP100). A three way mixed ANOVA model was used to analyze the iP100 amplitude with subject factors: electrode position (O1 or O2 and Oz), field stimulated (left or right) and eye stimulated (left or right). The iP100 amplitude of hemifield VEPs within one eye and across eyes were analyzed: Using a Bonferroni corrected t-tests for multiple comparisons. As with the full field, responses are displayed graphically with iP100 inter field difference (right hemifield iP100 minus left hemifield iP100 amplitude) plotted against each eye.

## 3. Results

**Figure 1:**
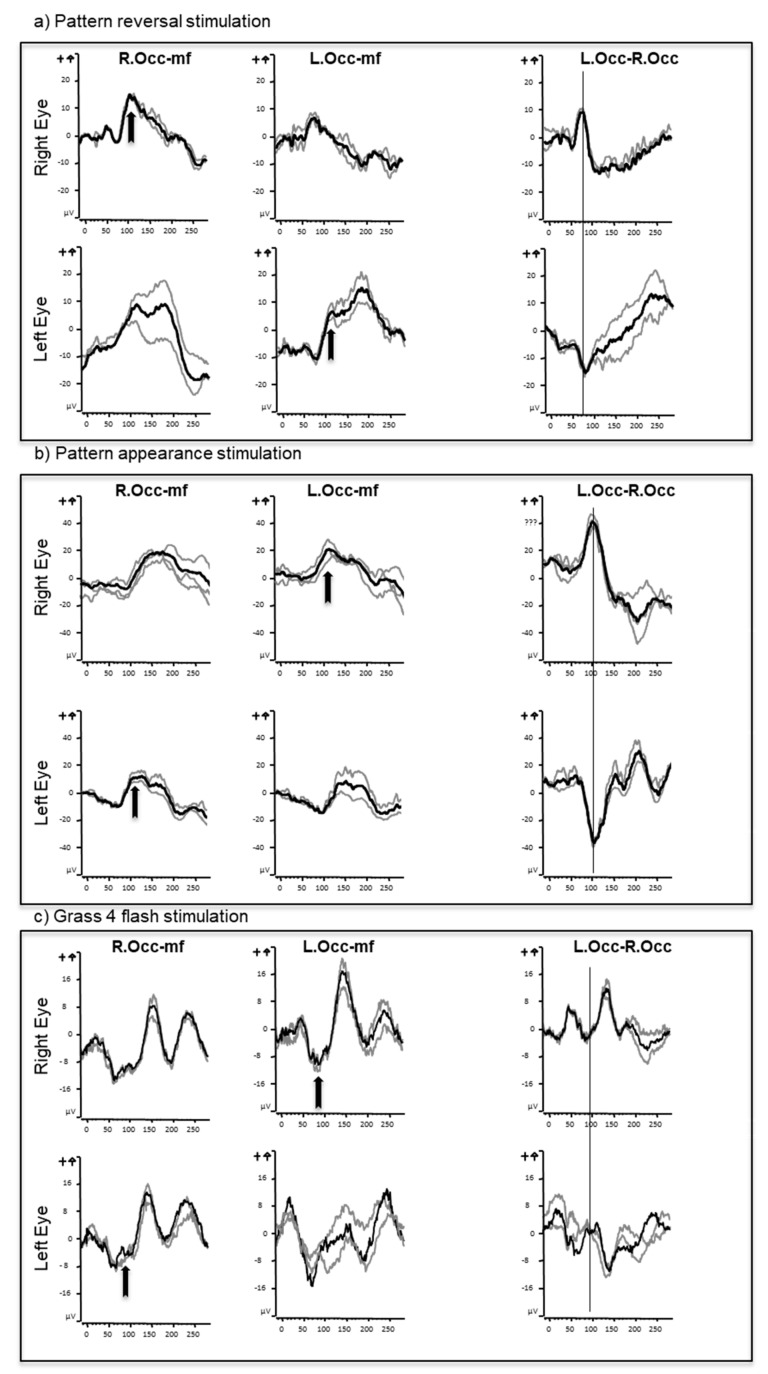
Full field pattern reversal (**a**), appearance (**b**), and flash (**c**) visual evoked potentials from one subject. Responses recorded from the right (R.Occ) and left (L.Occ) occiput from a mid-frontal (mf) reference. Gray lines represent individual trials, and black the grand average. Note the different patterns of crossed asymmetry between different stimuli. These are best seen with the subtraction waveform where responses from the right occiput are subtracted from the left (L.Occ–R.Occ). The crossing components are shown with arrows.

In all participants flash, FF pattern (onset and reversal) and HF pattern reversal stimulation evoked well-defined, reproducible VEPs. The stimuli tested in each subject are tabulated in Table 1, along with the type of trans-occipital asymmetry seen for each eye. Example waveforms are presented in Figure 1.

### 3.1. Full Field Pattern Reversal VEPs

**Figure 2:**
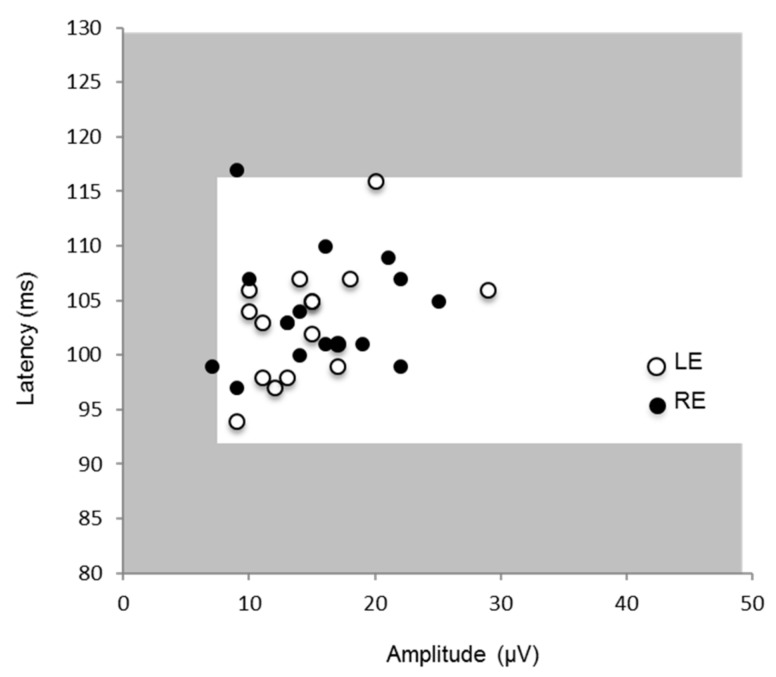
Amplitude and latency of P100 during monocular full field stimulation to pattern reversal test checks subtending 50 min of arc presented in a 28 degree field. The white area represents the 1st and 99th centile of the laboratories normal limits, whilst gray represents outside of normal limits. Responses for all eyes except two were within normal limits. LE: left eye; RE: right eye.

**Figure 3:**
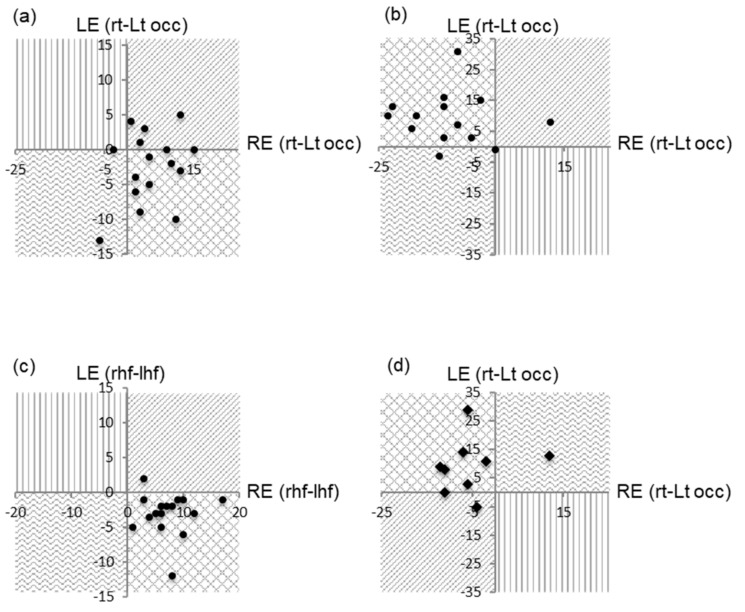
Comparison of monocular trans occipital amplitude asymmetries of the major VEP components. (**a**) Monocular full field pattern reversal responses, (**b**) hemifield responses, (**c**) pattern appearance, and (**d**) monocular flash.

As in controls, prVEPs in the albino patients were dominated by a positive component (P100) with a mean latency of 103 ms maximal at the midline (Table 2). The P100 at Oz was within normal limits for amplitude and latency in all participants for each eye; except two cases (Figure 2). Of these, one was minimally increased in latency and the other minimally decreased in amplitude. As a group the P100 measured 15.4 µV at 103 ms for the right eye and 14.8 µV at 103 ms for the left eye with no significant interocular difference in amplitude (*t* = 0.53, *p* = 0.60) or latency (*t* = 0.63, *p* = 0.53) (Table 2). Monocular reversal VEPs were asymmetrically distributed over the occiput for each eye in 16 cases (Figure 3a). In nine cases VEPs from each eye were larger over the occiput ipsilateral to the stimulated eye revealing a crossed asymmetry. In five cases both left and right eye VEPs were distributed homonymously, (larger over the right occiput in five cases and over the left in one case). In the remaining two cases responses were symmetrical in the left eye and asymmetrical in the right. Full field pattern reversal VEPs had a crossed asymmetry detection rate of 56.25% in this group (Table 1).

### 3.2. Full Field Pattern Appearance VEPs

FF paVEPs in this young age group consisted of a single positive component as a group measuring 24.6 µV at 109.2 ms for the right eye and 25.1 µV at 125.8 ms for the left eye. There was no significant interocular difference in the P1 amplitude (*t* = −2.16, *p* = 0.06) or latency (*t* = −0.38, *p* = 0.71). Monocular FF paVEPs were recorded in 9 of the 16 participants and were asymmetrical in all eyes except one (see Table 1, Figure 3c). 

In five patients, monocular FF paVEPs were larger over the occiput contralateral to the eye being stimulated revealing a crossed asymmetry. In three cases FF paVEPs were homonymously distributed over the right occiput, while in one case left eye FF paVEPs were symmetrical and right eye FF paVEPs were larger over the left occiput. The lowest crossed asymmetry detection rate of 55.55% was seen in FF paVEPs (Table 1). 

### 3.3. Flash VEPs

FVEPs were evident in all eyes and consisted of a positive component measuring as a group 23.9 µV at 117.1 ms for the right eye and 22.5 µV at 117.3 ms for the left eye. There was no significant interocular difference in the P1 amplitude (*t* = 0.60, *p* = 0.56) or latency (*t* = −0.9, *p* = 0.93). All monocular FVEPs were asymmetrically distributed. In 14 cases FVEPs had a greater negative-positive complex over the occiput contralateral to the eye being stimulated resulting in a crossed asymmetry. In the remaining two cases FVEPs were homonymously distributed for both eyes (larger over the right in one case and over the left in the other) (Figure 3d). FVEPs had the second highest crossed asymmetry detection rate of 87.5% (Table 1). 

### 3.4. Hemifield prVEPs

HF prVEP iP100 responses were recorded in all subjects. There was a significant interaction in the amplitude of the P100 between electrode position, eye stimulated, and hemifield stimulated (f(1,15) = 25.7; *p* = 0.00003). 

An effect was also evident for each eye individually when investigated employing a two-way repeated measure ANOVA (RE (f(1,15) = 26.0; *p* = 0.000132) and LE (f(1,15) = 5.2; *p* = 0.038)). These interactions were further investigated employing paired t-tests.

As expected the amplitude of the iP100 to stimulation of the crossing pathways were significantly larger than those to the non-crossing in each eye ((RE: *t* = 7.04, *p* = 0.000004), (LE: *t *= −3.94, *p* = 0.001)) (Figure 3b, Table 2).

Further analysis showed that stimulation of the crossing hemifield produced larger responses for the right eye compared to the left eye (RE-right HF, compared to LE-left HF) (*t* = 3.40, *p* = 0.004). Although there was a tendency for non-crossing pathway stimulation to evoke responses larger from the left hemisphere this did not reach significance (*t* = −1.47, *p* = 0.16). 

**Figure 4:**
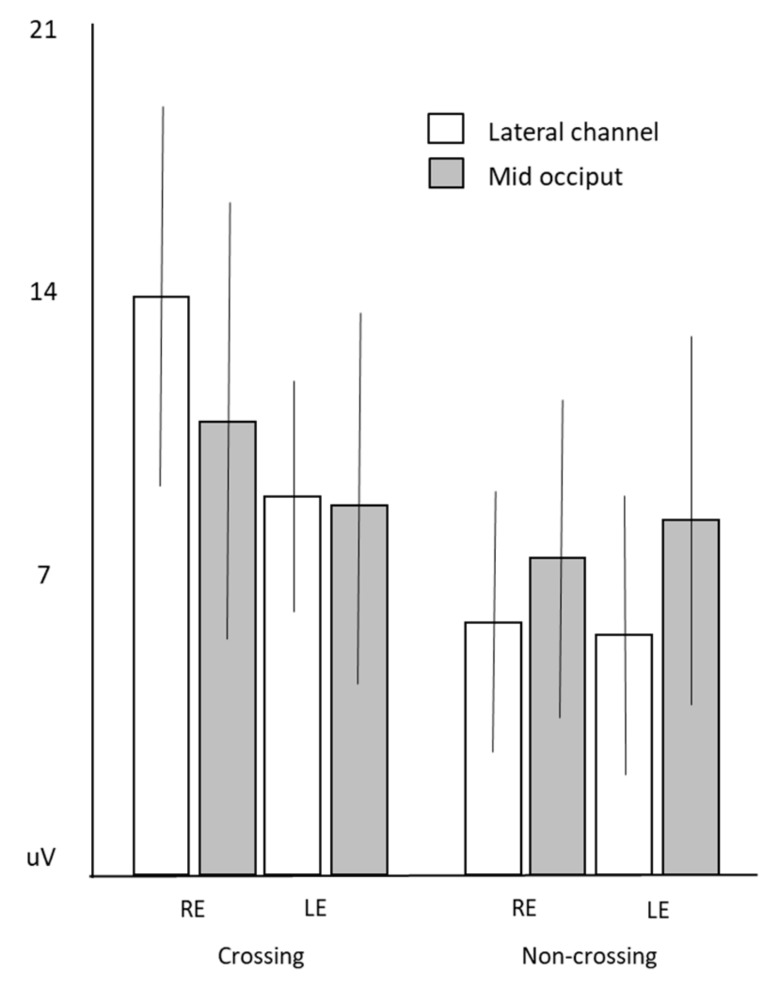
Mean amplitude of iP100 measured over the hemisphere ipsilateral to the field being stimulated, error bars are ± standard deviation. White bars measured over the ipsilateral channel and gray measured at mid occiput.

**Figure 5:**
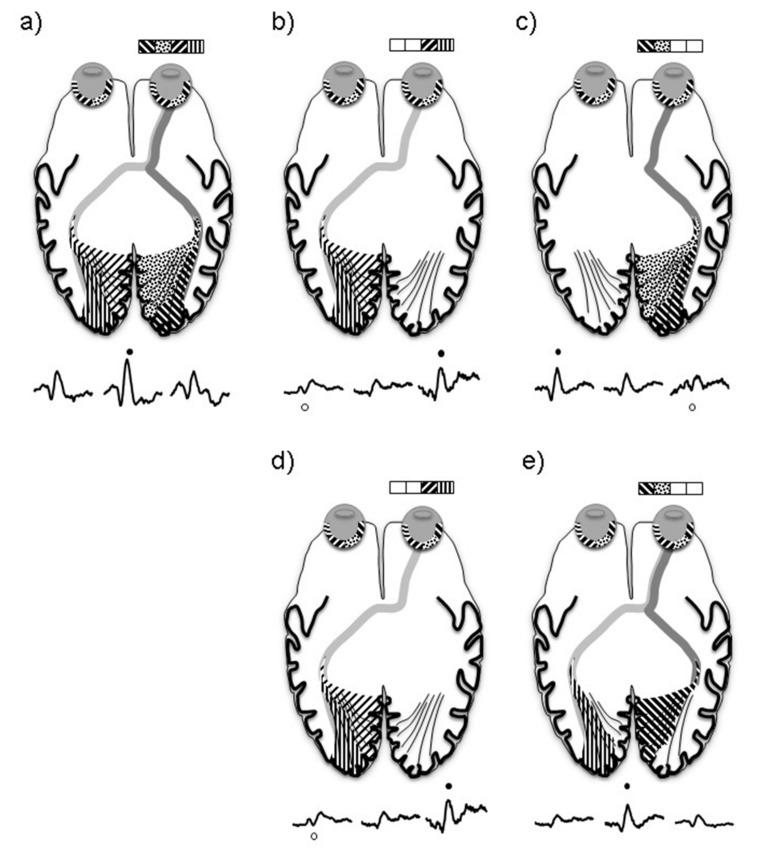
A schematic explaining the trans occipital distribution of a normal subject during monocular pattern reversal stimulation of the full field (**a**), right hemifield (**b**), and left hemifield (**c**). In comparison to in a subject with albinism when the right hemifield (**d**) and left hemifield (**e**) are stimulated. ● = P100, o = iP100.

The right eye, right HF prVEPs (due to the crossing pathway) showed an expected asymmetrical trans-occipital distribution with the iP100 significantly larger at the right occiput compared to the midline (*t *= 3.7; *p* = 0.02), while in comparison right eye left HF prVEPs were larger at the midline compared to the left occiput (*t *= −3.7; *p* = 0.002). In contrast the left eye left HF prVEPs (crossing pathway) showed a more symmetrical distribution about the midline compared to right eye right HF prVEPs, with no significant difference in the P100 amplitude at the mid or iP100 at the left occiput. The left eye right HF prVEPs were larger at the midline compared to the right occiput (*t *= −2.5; *p* = 0.024) (Figure 4, Table 2). These larger VEPs at the midline from stimulation of the non-crossing fields are represented for the right eye in Figure 5. Hemifield VEPs had the highest crossed asymmetry detection rate of 93.8%, as only one patient did not show a crossed asymmetry (Table 1).

## 4. Discussion

This group of individuals with albinism and steady fixation due to the lack of nystagmus, has given us the opportunity to investigate visual pathway function in response to hemifield stimulation employing electrophysiological techniques. 

As the major maturational of the prVEP P100 occurs within the first 12 months of life allowed we were able to analyze our cohort as a group, that would not have been possible if either flash or paVEP stimulation had been used alone. 

This study has revealed that the iP100 evoked by stimulation of the temporal field of albinoid subject are larger from the right eye compared to the left eye. This is suggested to be consistent with the larger left hemisphere responses due to cortical asymmetries as observed in normal subjects [10]. This was, however, unexpected considering the recent MRI studies that have revealed decreased cortical thickness and gyrification in albinos compared to controls, with a greater decrease observed in the left hemisphere compared to the right [4,30]. This could be due to the patients with albinism studied not being representative of albinism as a whole; perhaps those without nystagmus have a more typical cortical structure. 

Our findings suggest that the cortical differences observed between patients with albinism and controls have no electrophysiological consequence. 

In albinism the maximal amplitude VEP for left and right eye nasal field stimulation is recorded at the midline in contrast to control subjects where the largest VEP is lateralized over the hemisphere ipsilateral to the field stimulated. This reflects a cortical representation of the nasal field of patients with albinism in both right and left hemispheres (Figure 5e). Electrophysiologically the nasal retina pathways act similarly to that observed in control subjects. 

These findings can explain the differing pattern of trans occipital distribution between the left and right eye of the FF prVEPs in two cases (7 and 9). In both cases the left eye prVEPs, (dominated by right hemisphere activation), were symmetrical in distribution, i.e., maximal at Oz, and similar to the VEP produced by left HF stimulation in controls subjects due to the occipital petalia [10].

Despite at times ambiguous full field distributions, HF prVEP stimulation was found to be the most sensitive stimuli at detecting chiasmal misrouting associated with albinism without the confounding effects of nystagmus, evident in all, but one case in our series (13). In this case chiasmal misrouting however was detected in flash VEP, but not paVEPs. 

Although paVEPs had the lowest crossed asymmetry detection rate, a limiting factor interpreting this outcome is that paVEPs were tested in fewer patients, 9 of the 16, and as a result may not be a representative sample. In five cases above the age of seven paVEPs detected chiasmal misrouting. In two cases (4 and 5), although above the age of seven, misrouting was not detected by paVEPs and the remaining two cases (3 and 10) were below the age of seven with misrouting not detected. These findings are consistent with reports that the flash VEPs are more sensitive in detecting misrouting in infancy, while the C1 component of the paVEP is more sensitive after age seven years. No single type of stimuli detected the crossed asymmetry in all cases. This re-enforces previous recommendations that a combination of flash and pattern stimulation is needed to ensure mis-routing detection. It may also explain why in some people with albinism a VEP crossed asymmetry is not detected.

## 5. Conclusions

1. The electrophysiological pattern of VEP trans-occipital asymmetries seen in normal subjects due to the influence of asymmetric occipital petalia is also evident in our cohort of albinos without nystagmus. As evidenced by the HF VEP studies, we were able to demonstrate that stimulation of the crossing fields in albinos produces larger responses from the left hemisphere compared to the right, which is like the controls. This functional VEP similarity is notable given recent MRI studies that have revealed decreased gyrification and gray matter volume mainly in the left hemisphere of albinos compared to controls.
2. In cases where albinism is suspected, and no nystagmus is evident, hemifield stimulation should be attempted where possible as this study has shown it has superior detection sensitivity in detecting chiasmal misrouting.
3. Studies combining functional MRI imaging and electrophysiological studies in subjects without nystagmus are needed to further investigate interhemispheric structural and functional correlations of visual pathway misrouting in albinism.

## Figures and Tables

**Table 1:** Patients age, monocular visual acuity (LogMAR), and foveal grading. Gender and albinism type are also shown where OCA: oculocutaneous albinism, OA: ocular albinism, and HPS: Hermansky-Pudlak syndrome. The types of asymmetry detected by stimuli are represented by (∞), crossed asymmetry, (=), symmetrical, (→), larger over right, (←), larger over left. Where the asymmetry is different for each eye this is also recorded. The percentage detection of crossed asymmetry for each stimuli type is also shown. Note—patients 1, 4, 7, and 9 are two sets of twins.

	Age	Sex	RVA	LVA	Albinism Type	Fovea Grade	Hemi Field Reversal	Flash	Full Field Reversal	Full Field Appearance
**1**	7.80	F	0.36	0.38	OCA	3	∞	∞	∞	∞
**2**	7.77	M	0.32	0.56	OA	3	∞	∞	∞	∞
**3**	6.12	F	0.48	0.56	OCA	4	∞	∞	∞	→
**4**	9.43	F	0.38	0.66	OCA	4	∞	∞	∞	→
**5**	8.27	M	0.26	0.2	OA	4	∞	∞	∞	RE← LE =
**6**	13.61	M	0.25	0.3	OA	2	∞	∞	∞	
**7**	7.80	F	0.36	0.2	OCA	4	∞	∞	RE→ LE =	∞
**8**	4.81	M	0.4	0.45	OA	2	∞	∞	←	
**9**	9.43	F	0.34	0.2	OCA	4	∞	∞	RE← LE =	∞
**10**	5.85	F	0.44	0.56	OCA	3	∞	∞	→	→
**11**	6.81	F	0.3	0.3	OCA	2	∞	∞	→	
**12**	6.93	M	0.45	0.525	OA	4	∞	∞	→	
**13**	9.45	F	0.3	0.45	OCA	1	→	∞	→	
**14**	13.08	F	0.38	0.3	HPS	2	∞	→	∞	
**15**	7.75	M	0.575	0.55	OA	3	∞	←	∞	∞
**16**	5.1	F	0.3	0.6	OCA	1	∞	∞	∞	
Percentage detection rate of crossed asymmetry in total group (*n* = 16)							93.75%	87.5%	56.25%	
Percentage detection rate of crossed asymmetry in group that had all stimuli (*n* = 9)							100%	88.88%	66.66%	55.55%

F: Female; M: Male. LE: left eye; RE: right eye.

**Table 2:** The group average and standard deviation of amplitude and latency measures from either each eye and stimulus type. The half field responses evoked from the temporal field/crossing fibers of either eye are shown in bold.

	Right Eye				Left Eye			
	Amplitude ± SD (uV)		Latency ± SD (ms)		Amplitude ± SD (uV)		Latency ± SD (ms)	
	iP1	mP1	iP1	mP1	iP1	mP1	LE iP1	LE mP1
Reversal	11.6 ± 5.3	15.4 ± 5.3	103.9 ± 5.1	103.0 ± 5.1	10.0 ± 4.7	14.8 ± 5.0	103.0 ± 5.0	102.1 ± 6.1
Onset	18.0 ± 5.8	24.6 ± 6.1	112.4 ± 18.4	109.2 ± 8.2	18.9 ± 10.7	25.1 ± 8.0	114.0 ± 11.2	125.8 ± 11.4
Flash	15.5 ± 11.7	23.9 ± 11.8	115.1 ± 19.0	117.1 ± 20.6	14.5 ± 10.4	22.5 ± 10.9	118.1 ± 20.3	117.3 ± 20.0
R Half Field	12.3 uV ± 3.8	9.6 ± 4.6	100.7 ± 3.4	100.8 ± 4.8	6.1 uV ± 3.0	7.6 uV ± 3.7	104.1 ± 3.9	104.1 ± 3.9
L Half Field	5.1 uV ± 2.9	7.6 ± 3.4	106.44 ± 9.9	103.6 ± 10.6	9.3 uV ± 2.7	9.1 uV ± 2.59	99.6.1 ± 6.1	99.6.1 ± 6.1

SD: standard deviation.
